# How Does the Extraction of Local and Global Auditory Regularities Vary with Context?

**DOI:** 10.1371/journal.pone.0107227

**Published:** 2014-09-08

**Authors:** Sébastien Marti, Louis Thibault, Stanislas Dehaene

**Affiliations:** 1 INSERM, U992, Cognitive Neuroimaging Unit, Gif/Yvette, France; 2 CEA, DSV/I2BM, NeuroSpin Center, Gif/Yvette, France; 3 Collège de France, Paris, France; 4 Laboratoire Psychologie de la Perception, UMR 8242, Université Paris Descartes, Paris, France; Harvard Medical School/Massachusetts General Hospital, United States of America

## Abstract

How does the human brain extract regularities from its environment? There is evidence that short range or ‘local’ regularities (within seconds) are automatically detected by the brain while long range or ‘global’ regularities (over tens of seconds or more) require conscious awareness. In the present experiment, we asked whether participants' attention was needed to acquire such auditory regularities, to detect their violation or both. We designed a paradigm in which participants listened to predictable sounds. Subjects could be distracted by a visual task at two moments: when they were first exposed to a regularity or when they detected violations of this regularity. MEG recordings revealed that early brain responses (100–130 ms) to violations of short range regularities were unaffected by visual distraction and driven essentially by local transitional probabilities. Based on global workspace theory and prior results, we expected that visual distraction would eliminate the long range global effect, but unexpectedly, we found the contrary, i.e. late brain responses (300–600 ms) to violations of long range regularities on audio-visual trials but not on auditory only trials. Further analyses showed that, in fact, visual distraction was incomplete and that auditory and visual stimuli interfered in both directions. Our results show that conscious, attentive subjects can learn the long range dependencies present in auditory stimuli even while performing a visual task on synchronous visual stimuli. Furthermore, they acquire a complex regularity and end up making different predictions for the very same stimulus depending on the context (i.e. absence or presence of visual stimuli). These results suggest that while short-range regularity detection is driven by local transitional probabilities between stimuli, the human brain detects and stores long-range regularities in a highly flexible, context dependent manner.

## Introduction

The human brain has the ability to extract patterns or regularities in its environment, e.g. object A is always followed by object B but never by object C. The learning of regularities is a key process in various cognitive domains, for instance in the learning of motor sequences [1], in the guidance of attention to behaviorally relevant objects [2], in language development in infants [3]. In the auditory domain, regularities have also been proposed to serve as perceptual objects [4]. However, much remains unknown about the neural processes by which regularities are extracted and used to predict future events.

There is evidence that the brain can detect transitional probabilities in an automatic way, i.e. even when the subject's attention is distracted [5], [6] or when stimuli are presented below the threshold of awareness [7]. An observer might not be aware of the acquired knowledge but still be able to use it non-consciously [8], [9]. Automatic brain responses to a violation of a rule (or regularity) can also be detected if the stimuli are in close or local temporal vicinity (i.e. within few seconds). M/EEG studies have shown that an early mismatch response (the mismatch negativity, MMN) [10] is observed even if subjects are distracted [11], [12], [13], [14] or unconscious [12], [13], [15], [16], [17], [18], [19], [20], [21] although it can be modulated by attention [11], [14], [22]. The MMN can be produced with complex sequences such as a melody or a rhythm in both attentive and distracted situations [23], [24]. It is even possible to observe an MMN when predictions are between auditory features (e.g. duration and pitch of stimuli) and the subjects may not be necessarily aware of these complex rules [8]. There is also evidence that this brain response is mainly driven by regularities presented within a short temporal scale as its amplitude quickly decreases when the delay between stimuli increases [25], [26], [27]. Theoretical and neuronal models of the MMN proposed that it originates from the activity of neurons signaling a mismatch between the new incoming sensory information and the mnesic representation of recent events [28], [29], [30], [31], [32], [33]. Taken together, these results suggest that the ability of the human brain to extract rules non-consciously might be restrained to a short temporal range, sensitive to the temporal relations between stimuli presented within few seconds [32], [33].

The brain can also extract regularities on a longer time range. The global sequence of stimuli, over tens of seconds or minutes, can influence the response to deviants [34]. However, the properties of the brain responses are drastically different. EEG recordings revealed that violations of a long-range regularity induce a late and sustained positivity over parietal and central electrodes, the so-called P300. The P300 is strongly sensitive to manipulations of the subject's attention [35], [36], [37], and is intimately related to conscious awareness [38], [39], [40].

An experimental paradigm allowing orthogonal manipulations of the MMN and the P300 components was recently designed [12]. This ‘local-global’ paradigm is based on an auditory oddball paradigm in which a sequence of sounds is presented at each trial. The sequence is composed of a standard sound repeated a certain number of time, followed by a deviant sound (e.g. XXXXY). The comparison to a condition in which all the sounds of the sequence are standard (e.g. XXXXX) typically reveals the occurrence of the MMN. Crucially, if the deviant sequence XXXXY is highly frequent within a block (e.g. 80% of the trials are XXXXY and 20% are XXXXX), the subjects would expect the last sound to be deviant. The sequence XXXXY is thus standard at the *global* level (i.e. over the experimental block) and deviant at the *local* level (i.e. within a single trial). The local standard sequence XXXXX becomes a global deviant and triggers the occurrence of a P300 component. In sum, the *local-global paradigm* is a 2×2 which allows orthogonal manipulations of automatic versus conscious brain responses to regularity violations.

The global neuronal workspace theory of consciousness (GNW) proposes that the P300 is related to the ignition of a distributed parieto-frontal network which would sustain and broadcast the sensory information to multiple brain systems [41], [42], [43]. Conscious processes would maintain the stimulus-related information available as long as the subject's attention is focused on it. This would allow the participant to compare stimuli on a much longer time range, possibly over minutes. The comprehension of the global structure behind the stimulus sequence might thus require consciousness. In other words, the GNW theory predicts that the local effect triggers automatic MMN responses, driven by transitional probabilities inferred from the close temporal neighborhood. By contrast, the global effect triggers conscious P300-type responses, driven by global stimulus properties inferred from a longer time scale. Thus, a key notion in this distinction is the temporal span needed to infer the regularity which solicits two different brain processes.

The brain response to the global violation depends on the subject's attention. Bekinschtein and colleagues (2009) showed that distracting subjects' attention precluded the appearance of a P300 to global deviants. Interestingly, debriefing subjects revealed that only subjects who could attend to the sounds, and in which a P300 could be measured after global violations, were aware of standard and deviant stimuli. This suggests that the P300 component was observed only when subjects could attend to the sound and were aware of the global regularity and of its violations. Further research with coma and vegetative state patients further consolidated the link between the global P300 response and conscious detection [12], [13], [44].

The Bekinschtein et al. (2009) data leave open the exact reason why inattention disrupts global novelty detection. It is impossible to know from these data if attention is needed to detect the global regularity, to make predictions for new stimuli, or both. It could be that global regularities are learned unconsciously and without attention, but that attention is needed to detect their violation. Alternatively, it could be that attention is needed to acquire the regularity in the first place, but that once it is acquired, its violations are automatically detected. Finally, attention could disrupt both learning and violation detection.

We addressed this question in the present experiment by manipulating attention independently at two moments: while subjects were first exposed to a regularity (exposure phase), and while subjects were using the rule to make predictions and detect violations (test phase). The GNW theory predicts a sharp contrast in brain responses to local and global violations. When the subject's attention is focused on another task, only brain responses to local violations should be observed. To anticipate on our results, we found unexpectedly a brain response to global violations even when subjects were occupied by another task. Interestingly however, this response varied depending on the presence of visual stimuli. We conclude that the brain was actually not fully distracted, remained able to attend to both visual and auditory stimuli, and was even able to extract and maintain two different auditory rules depending on whether the visual stimulus was present or not.

## Method

### Subjects

Twenty-two adults (6 women) aged between 21 and 29 years old (mean age: 24 years) participated in the study. The study was approved by the “Comité de Protection des Personnes” and all participants gave informed and written consent before testing and received a compensation of €80 after their participation. All were naïve with respect to the task and had normal or corrected to normal vision.

### Experimental design

#### The auditory “local-global” paradigm

**Figure 1:**
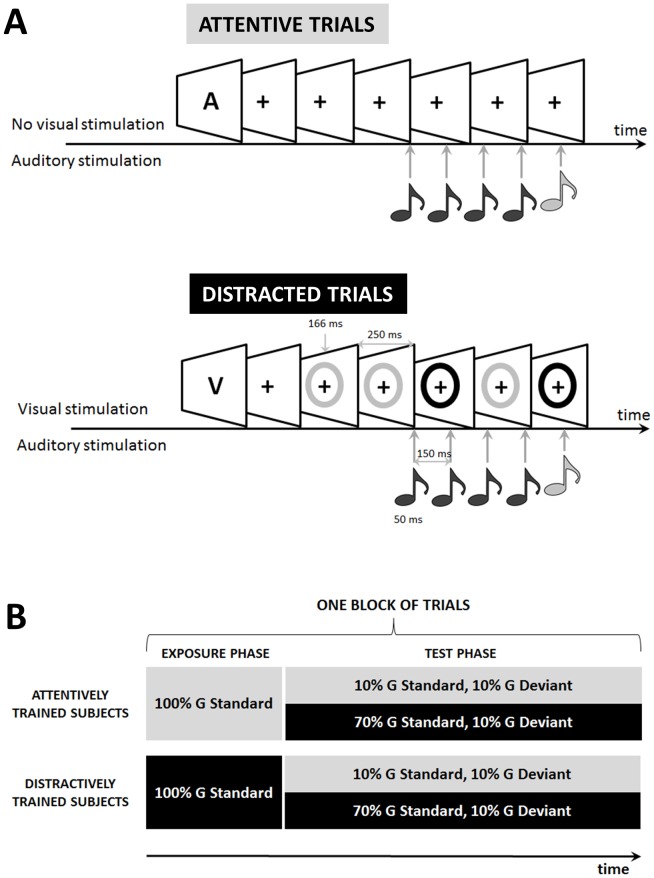
Schematic representation of the paradigm. (A) A trial started with the presentation of a visual cue “A” for auditory only trials (‘attentive’ trials) or “V” for audio-visual trials (‘distracted’ trials) (top and bottom respectively). For auditory only trials, subjects had to pay attention to a sequence of five sounds (duration: 50 ms, SOA: 150 ms). The fifth sound could be either standard or deviant according to a local (within a second) or a global (over tens of seconds) regularity (see method). For audio-visual trials, five colored rings (duration 166 ms, SOA: 250 ms) were presented concurrently to the sounds and the subject was instructed to estimate which of two colors was presented more often. (B) Schematic representation of a block of trials. An experimental block started with an ‘exposure phase’ in which only global standard stimuli were presented so that the participant could learn the regularity with perfectly predictable sounds. Half of the subjects were distracted during the exposure phase (100% distracted global standard trials, represented in black) while the other half was not (100% attentive global standard trials, represented in grey). We named these groups of subjects ‘distractively trained (AT) subjects’ and ‘attentively trained (DT) subjects’ respectively. The exposure phase was followed by a ‘test phase’ composed of 80% global standard stimuli and 20% global deviant. Attentive and distracted trials were inter-mixed during the test phase: 70% of global standard and 10% of global deviant were distracted trials, leaving an equal proportion of global standard and deviant in attentive trials (10% each).

Participants listened passively to series of five pure tones (50 ms duration sinusoidal tones with 7 ms rise and fall times). Tones frequencies could be either 700 Hz or 1400 Hz (X and Y sounds respectively). Stimuli were separated by a 150 ms stimuli onset asynchrony (SOA). At the trial level, the first four sounds in a series were identical and the last one could be either identical (e.g. XXXXX) or different (e.g. XXXXY), respectively called *local standard* and *local deviant* conditions (figure 1A). At the block level, a randomly selected series was presented 80% of the trials (*global standard* condition), and another series was presented 20% of the trials (*global deviant* condition). Critically, the local (i.e. trial-level) and global (block-level) rules were orthogonal so that, for instance, the fifth stimulus of a series could be simultaneously local standard and global deviant (i.e. 80% of XXXXY sequences and 20% XXXXX). Each series of sound served as the standard stimulus for one type of block. Each type of block was presented twice, for a total of eight blocks. For the purpose of our experiment, X and Y sounds were not related to any task. However, in order to ensure that subjects were paying attention to the sounds, we asked them to detect a target stimulus (T), a burst of Gaussian white noise (50 ms duration), occasionally presented at the end of a series (e.g. XXXXT) by pressing a response button as fast as possible. Four target trials were presented within a block.

#### Exposure phase and test phase

An experimental block started with the presentation of 25 global standard stimuli (“Exposure phase”) so that the participant had the opportunity to learn the regularity with sounds that were perfectly predictable. Critically for the present experiment, we wanted to test whether subjects' attention was needed to learn the regularity. Thus, half of the subjects were distracted by a visual task (see below) during the exposure phase (distractively trained subjects, DT) while the other half were not (attentively trained subjects, AT, see figure 1B). Specifically, the AT subjects performed 25 trials with only auditory stimuli and, separately, 25 trials with only visual stimuli. Unbeknownst to the subject, this exposure phase was followed by a “test phase” composed of 80% of global standard trials and 20% of global deviant trials. One objective was to test whether attention was needed for the detection of a global deviant stimulus. Thus, during the test phase, all subjects had to perform a demanding visual task, the hypothesis being that global deviancy would not be detected in these trials. This manipulation was simply aimed to replicate Bekinschtein and colleagues' results that no global effect is observed when subjects' attention is distracted [12]. If subjects were able to learn the regularity while being distracted, then during the test phase we should find a difference in the amplitude of late brain activations in response to global deviant versus global standard for both DT and AT subjects. On the other hand, if attention is needed for subjects to learn the global regularity, then only AT subjects should show a difference between global standard and deviant in attentive trials. Importantly, if the proportion of global standard and global deviant was still imbalanced in attentive trials, subjects would simply learn the regularity during these trials, making the exposure phase obsolete. Therefore, the proportions of global standard and global deviant were equalized in attentive trials. To sum up, the test phase was composed of attentive trials with equal proportion of global standard and global deviant (10% each), and of distracted trials with imbalanced proportion of global standard and global deviant (70% and 10% respectively, see figure 1B).

#### The visual distraction task

We used a visual distraction task presented in parallel to the sounds to manipulate the amount of attentional resources devoted to the sounds (figure 1A). A trial started with a 350 ms warning screen during which the letter ‘V’ was presented, indicating that the trial included a visual task (as opposed to ‘A’ when only sounds were presented). A fixation cross was then displayed and remained on the screen until the end of the trial. 400 ms after the onset of the fixation cross, five colored rings (yellow or blue) were presented in a pseudo-random sequence (duration: 166 ms, SOA: 250 ms, figure 1A). The number of blue and yellow stimuli differed by one and participants were instructed to determine which color appeared more frequently by pressing a button with their right thumb (blue) or with their right index (yellow) as fast as possible. The first auditory stimulus was presented 250 ms after the first colored ring. After the offset of the last stimulus the fixation cross remained on the screen for a variable delay (700–1000 ms) before the beginning of the next trial.

### Apparatus

Stimuli were back projected (refresh rate: 60 Hz) on a screen placed 60 cm in front of the subject under standard overhead fluorescent lighting. The sequence was controlled by a Pentium IV PC running E-Prime 1.1 software (PST Inc.). Sounds were presented through non-magnetic earphones. The sound intensity was constant across subjects and set to be comfortable. None of the subjects reported any problem hearing the sounds. We used a five button non-magnetic response box (Cambridge Research Systems Ltd., Fibre Optic Response Pad) to record their motor responses.

### MEG recordings

While subjects performed the cognitive tasks, we continuously recorded brain activity (sampling rate: 1000 Hz) using a 306-channel whole-head magnetometer (Elekta Neuromag) inside a magnetically shielded room (Maxshield) to decrease electromagnetic noise. Channels were organized in 102 triplets, each one composed of a magnetometer and two orthogonal planar gradiometers. Four head position indicators were placed over frontal and mastoïdian skull areas. The subject's head position was then measured at the beginning of each run using an isotrak polhemus Inc. system to compensate for head movements. Horizontal and vertical electro-oculograms and electrocardiogram were recorded simultaneously for offline rejection of eye movements and cardiac artefacts.

Signal Space Separation (SSS) method was applied to decrease the impact of external noise and sensor artefacts by separating the magnetic fields arising from sources inside the sensor helmet and those arising from sources outside [45]. MEG signals were low-pass filtered at 330 Hz. Gradiometers and magnetometers with amplitudes continuously exceeding 3000 fT/cm^2^ and 3000 fT respectively were set as bad channels and excluded from further analysis. SSS correction, head movement compensation and bad channels correction were applied using the MaxFilter Software (Elekta Neuromag). Continuous data were then epoched using Fieldtrip software (http://fieldtrip.fcdonders.nl/). Trials were time locked to the onset of the last sound with a time window starting 1.1 sec before its onset (i.e. 500 ms before the first sound) and ending 0.9 sec after. Each trial was baseline corrected using the first 250 ms of the epoch. The variance of the MEG signals across sensors was computed for each trial and displayed in a scatter plot. This variance was used as an index to visually inspect and reject trials that might be contaminated by muscles or movement artefacts. We used principal component analyses (PCA), applied separately for each type of sensor, to identify and reject artifacted components of the MEG signal. The continuous recordings of the ECG and EOG were epoched timelocked to the peak of the QRS complex for the ECG and to the peak of blink artefact for EOG before being subjected to PCA decomposition. The components related to artifacts were then projected out from the raw data. No more than two components for each type of artefact were rejected from the MEG data.

### Statistical analyses

To examine differences between experimental conditions, data were low pass filtered at 30 Hz and a two-tailed paired t-tests was performed at every sensor and every time sample (the threshold was set at p = 0.05). A correction for multiple comparisons was then applied using cluster-based permutation tests as implemented in the fieldtrip software. Although paired t-tests were parametric, the correction for multiple comparisons implemented in Fieldtrip is a non-parametric clustering method [46]. For every time sample, all sensors whose t-values exceeded the threshold were selected and clustered on the basis of spatial adjacency. On average 13 sensors were included in a cluster with a minimum of two sensors passing the threshold to form a cluster. A cluster-level statistic was then computed by taking the sum of the t-values within every cluster. This operation was repeated 1000 times on random partition of the data (Monte Carlo method) to compute the significance probability. The final corrected threshold was set at p = 0.05. Statistical analyses were performed on a time window from 50 to 250 ms and from 50 to 700 ms for the local effect and global effect respectively. These time windows were defined a priori, based on previous studies using similar local-global paradigms [12], [33]. The statistical analyses were performed separately for longitudinal gradiometers, latitudinal gradiometers and magnetometers given the different nature of these types of sensors. Finally, given that in distracted trials the proportion of global standard and global deviant was strongly imbalanced (70% and 10% respectively), we selected 10% of the global standard stimuli for comparison purposes, with the condition that it did not follow a global deviant.

We also conducted peak-to-peak measurements for a more detailed analysis of the influence of experimental factors on ERFs amplitude. We computed the difference in amplitude between two peaks of the ERFs (local effect: mean amplitude between 50 and 150 ms minus mean amplitude between 150 and 250 ms; global effect: mean amplitude between 100 and 200 ms minus mean amplitude between 350 and 600 ms) for a representative group of sensors. The experimental conditions were then compared in a repeated-measures ANOVA with Stimulus type (deviant versus standard) and Trial type (attentive versus distracted) as within-subject factors and Group type (AT versus DT subjects) as a between-subject factor.

### Effect-matched spatial filtering

The analyses of auditory ERFs raised new hypotheses regarding the brain activity specifically related to the visual stimuli. In order to examine visual ERFs, we used a recently developed spatial filter method (“effect-matched spatial filtering” [47]) rather than arbitrarily selecting a group of sensors over visual areas. This method has the advantage to be data-driven and to increase substantially the signal to noise ratio. The first step was to subtract the mean ERFs in attentive “auditory only” trials from the mean ERFs in distracted “audio-visual” trials, resulting in brain activity mainly driven by the visual task. We then selected a late component (500–650 ms) related to the onset of the first colored ring (well before the potentially deviant sound). The average topography of this component was used as a spatial filter. Each trial was projected on this subspace using a leave-one-out cross validation procedure (see Schurger et al., 2013 for details of the method). After applying this filter to each epoch we were able to test whether the presentation of the global deviant sound would affect visual processing. We used paired t-tests to compare the amplitude of the ERFs between conditions with a criterion of p<0.05 for at least 30 consecutive time samples.

## Results

### Behavioral results

The performance at the visual task was not significantly different between AT and DT subjects (76% and 80% respectively). This finding shows that for both groups the task was quite difficult and required subjects to focus on the visual stimuli. It is however possible that the presentation of a local or global auditory deviant stimulus would interrupt the visual task. To evaluate this possibility, we examined whether the auditory experimental conditions interfered with the performance of the visual task on the same trial. Results from a repeated-measures ANOVA with a between-subjects factor (AT versus DT subjects) and auditory conditions as within-subjects factors did not reveal any effect or interactions of between- and/or within-subjects factors, neither for visual hit rate nor for reaction time. These results suggest that participants mainly focused on the visual task and managed to avoid being distracted by the concurrent auditory stimuli.

### The detection of local violations is unaffected by the visual task

**Figure 2:**
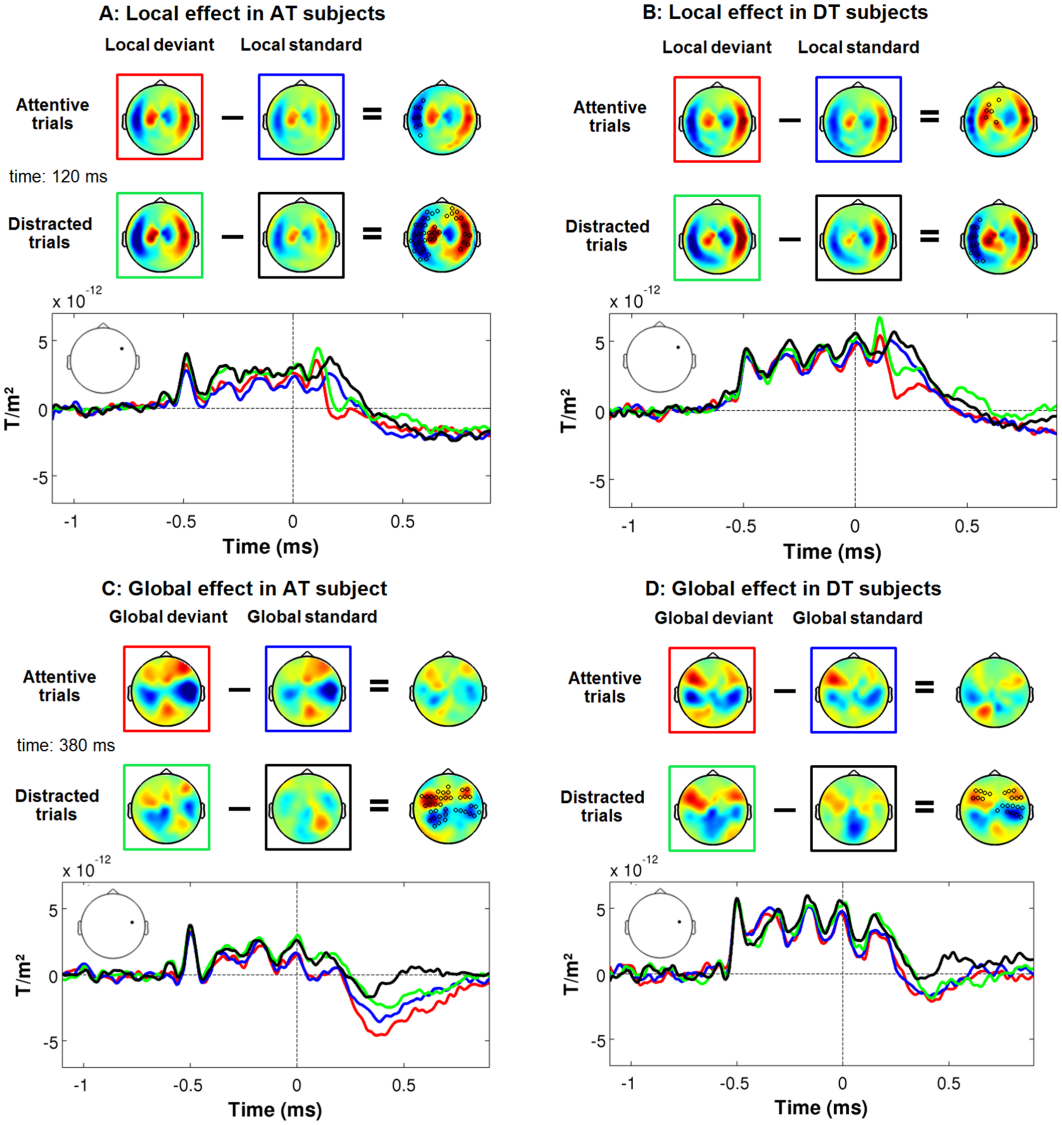
ERFs related to local and global violations. Group averaged event-related fields for local (A, B) and global (C, D) violations in AT (A, C) and DT (B, D) subjects. In each panel, the first line of topographies represent deviant, standard and the difference between deviant and standard at 120 ms for the local effect and 380 ms for the global effect in attentive trials. The second line of topographies represents the same subtraction but for distracted trials. Colored squares indicate the corresponding time course depicted below the topographies. Black circles on topographies represent channels showing a significant difference between standard and deviant conditions (see method). Finally, a representative channel shows the time course of the deviancy effect both in attentive (blue and red lines) and distracted trials (green and black lines). As can be seen, local violations induced a mismatch effect in the ERFs which remained essentially unaffected by the visual task. However, a global effect was observed only on distracted trials. In attentive trials, both standard and deviant induced large late ERFs.

**Figure 3:**
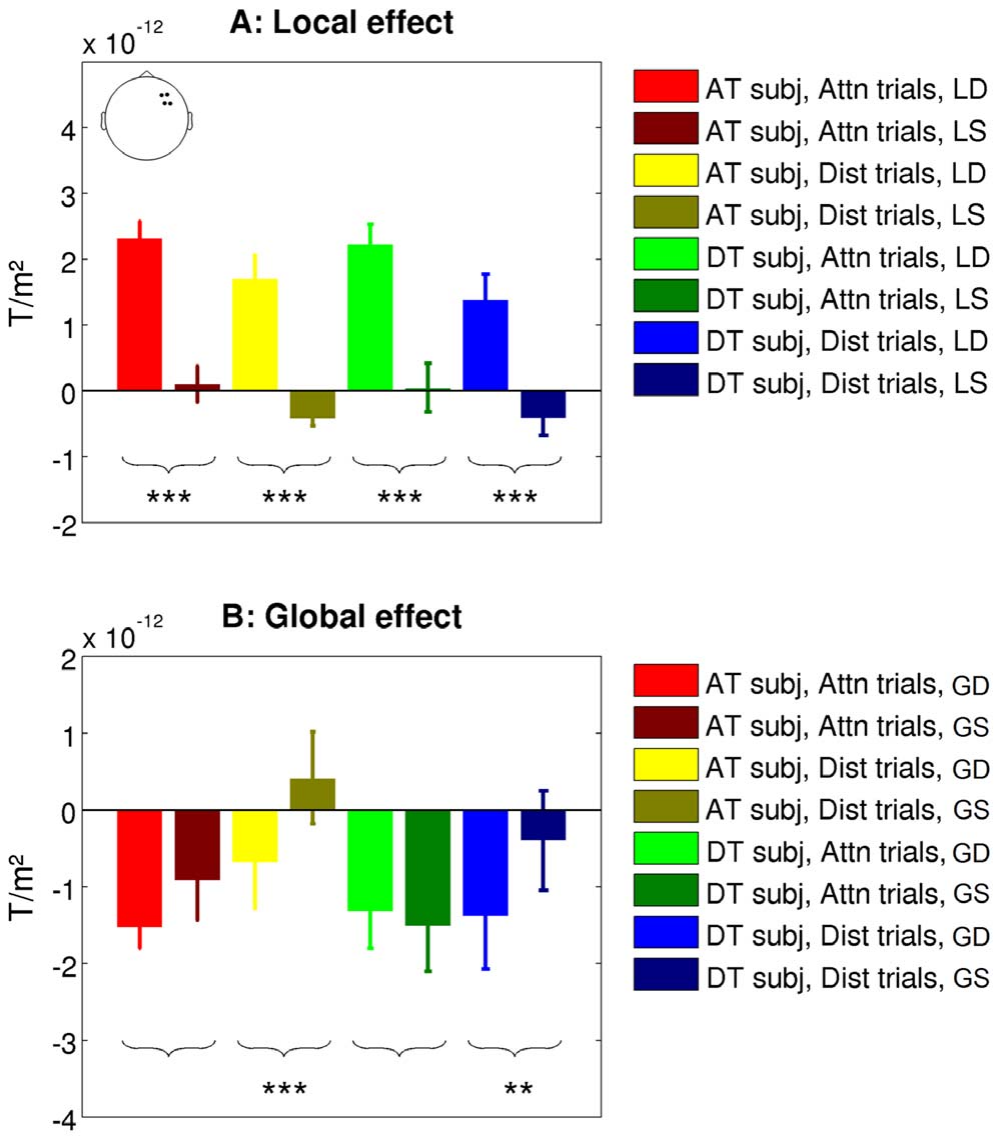
Mean amplitude (± s.e.m.) for local (A) and global (B) effects. For the local effect, we selected four right temporal sensors and measured the difference in amplitude between two peaks of the ERFs: we averaged the amplitude between 50 and 150 ms and between 150 and 250 ms and computed the difference between the two. The same procedure was used for the global effect but with a different set of sensors (as indicated by the empty head inset) and different time windows: 100–200 ms and 350–600 ms. Each color represents a combination of group and condition: attentive (red) and distracted (yellow) trials in AT subjects; attentive (green) and distracted (blue) trials in DT subjects. Dark and bright colors represent standard and deviant stimuli respectively. Brackets and stars represent results from t test comparing amplitudes for deviants and standard stimuli: *: p<0.05; **: p<0.01; ***: p<0.001.

When the fifth sound of a sequence differed from the four preceding ones, thus violating the local regularity, we observed a divergence in the event-related magnetic fields (ERFs) around ∼100–130 ms. This effect was observed for both groups of subjects and in both attentive and distracted trials (figure 2A and 2B). Scalp patterns of this mismatch effect revealed a significant difference between local standard and local deviant stimuli essentially in temporal sensors bilaterally. A repeated-measure ANOVA performed on peak amplitude measurements (see method) revealed main effects of Stimulus type (F(1,10) = 88.78, p<0.001) and Trial type (F(1,10) = 18.57, p<0.01) but no interactions between these factors (figure 3A). These results show that while the amplitude of the ERFs for local deviant and local standard stimuli was slightly reduced on distracted trials compared to attentive trials, the mismatch response itself was unaffected by experimental manipulations. This is consistent with previous studies which showed that local deviancy can be processed in an automatic manner, independently of attention [48], [49].

### The detection of global violations depends on the context

One of our predictions was that the violation of the global rule would produce late and sustained brain activations in AT but not DT subjects. Surprisingly, we found instead that in both groups of subjects, global standard and global deviant induced similar late activations which did not differ significantly between them (figure 2C–D). In addition, a significant effect of global violation was observed on distracted trials. A repeated-measure ANOVA on peak amplitude measurements (figure 3B) revealed main effects of Stimulus type (F(1,10) = 18.41, p<0.01) and Trial type (F(1,10) = 5.54, p<0.05), and an interaction between the two (F(1,10) = 9.54, p = 0.01). Contrast analyses revealed that global deviants induced ERFs with a larger amplitude only on distracted trials (t = −15.06, p<0.001 and t = −4.28, p<0.01 respectively for AT and DT subjects), although we also observed a trend on attentive trials for AT subjects (t = −2.03, p = 0.07).

#### Global effect on attentive trials

The absence of a global effect (i.e. a difference between global standard and global deviant) on attentive trials can be puzzling at first sight. However, an important feature of our experiment can explain these results: on attentive trials, the proportions of global standard and deviant stimuli were equivalent. This property of the design was adopted to ensure that subjects could not learn the auditory regularity on the attentive trials of the test phase. However, it implied that a higher-level rule could be learnt: subjects could learn that, when no visual stimuli are presented, global standard and global deviant are equiprobable.

**Figure 4:**
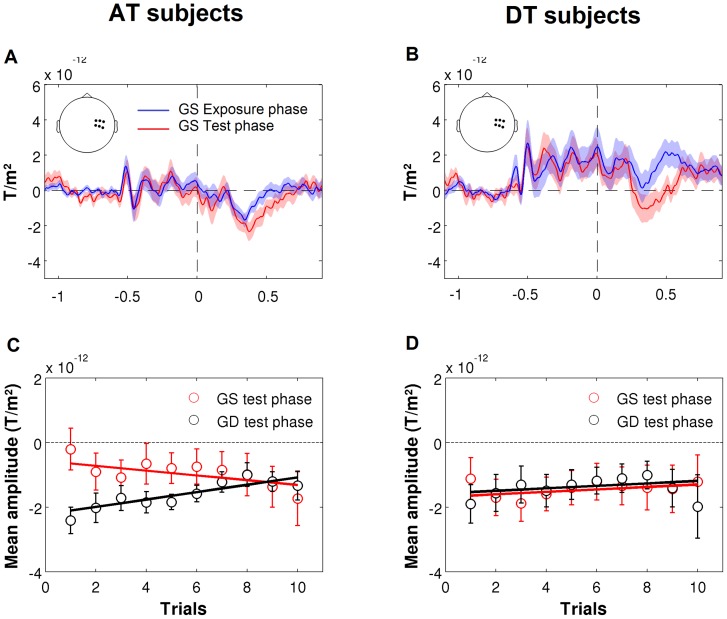
Temporal evolution of the global effect on attentive trials at the beginning of the test phase. Panels A and B represent an average of six sensors showing ERFs following the global standard stimulus during the exposure phase (blue) and during the test phase (red) for AT subjects (A) and DT subjects (B). The amplitude of late ERFs increased from the exposure phase to the test phase. Panel C and D represent the mean amplitude across subjects for the same sensors and time windows as in figure 3B for trials 1 to 10 (for display purposes, data points were smoothed using a moving average with a window of 1). During the test phase, the amplitude of late ERFs progressively increased for global standard stimuli (red) and decreased for global deviant (black) in AT subjects (C). By contrast, the amplitude of ERFs in DT subjects remained stable along the test phase (D), showing that the brain responses to global standard and deviant in that phase never differed.

The results depicted in figure 4 support this interpretation. The above hypothesis predicts that AT subjects should apply the rule learnt in the exposure phase at the beginning of the test phase, but then they should quickly learn that this rule is no longer true for attentive trials where no visual stimuli are present. In other words, we should see a global effect at the beginning of the test phase but not at the end. Indeed, a direct comparison of ERFs in the exposure phase and in the test phase (attentive trials) revealed a significant increase in gradiometer amplitude 350–600 ms after a global standard stimulus. Figure 4A shows the time course of this effect on right temporal sensors. In addition, for each subject, we measured a peak-to-peak difference in amplitude for the first ten trials of the test phase. We found that the gap in amplitude between the global standard and the global deviant progressively shrank along the test phase (figure 4C): for the global standard stimulus, the amplitude of the ERFs tended to increase from the beginning to the end of the experimental block (mean slope of a linear regression with trial position: −7.4e^−14^±5e^−14^, t = −1.45, p = 0.2) while it decreased for the global deviant stimulus (mean slope: 1.14e^−13^±4e^−14^, t = 2.79, p<0.05). This suggests that AT subjects actually applied the global auditory rule and showed an effect of global violations at the beginning of the test phase but then quickly learnt that sounds were unpredictable when presented in “auditory only” trials.

Regarding DT subjects, a direct comparison between the exposure and test phases for the global standard was impossible because there was no “auditory only” trials in the exposure phase for these subjects. Still, as can be seen in figure 4B, the time course of activity averaged over temporal gradiometers suggests a difference in amplitude between the exposure and the test phases in response to the global standard stimulus. The examination of single-trials revealed however a pattern different from AT subjects: the amplitude of late ERFs remained stable along an experimental session (mean slopes of linear regressions were not significantly different from 0) and did not reveal an “adaptation” pattern as the one observed in AT subjects (figure 4D). This suggests that DT subjects either were not able to learn the rule or did not apply it on attentive trials.

#### Global effect on distracted trials

Our second prediction was that when the participant's attention is monopolized by the visual task, we should not observe any effect of global deviancy. Contrary to what we expected, we did measure a significant difference between the global deviant and the global standard stimuli in both groups of subjects (figure 2C–D).

This finding suggests that, contrary to Bekinschtein et al's (2009), our visual distraction paradigm with synchronous auditory and visual stimuli was not fully successful in removing any attention to the auditory stimuli. In detail, three hypotheses can explain our results. First, there might be a competition between auditory and visual stimuli; subjects' attention may not be fully dragged to the visual stimuli, and it may be especially attracted to the auditory stimulus when those deviate from the expected pattern. In this case, we should predict that the late brain activity evoked by global auditory deviants would interfere with the execution of the visual task possibly via a bottleneck effect [50], [51]. Second, there might be a cooperation between auditory and visual stimuli: subjects might have learnt that these stimuli are always synchronized, detected their temporal relationships, and built a new audio-visual chunk [52] which is violated on global deviant trials. Third, resources devoted to vision and audition might be partially independent. In that case, the detection of deviant sounds would be possible during distracted trials at least to some extent, and the brain responses to auditory and visual stimuli should not interfere with each other.

The first two hypotheses predict potentially slower reaction times after the presentation of a deviant sound while the last one predicts no effect of the deviant sound on the response time. As mentioned earlier, we found no evidence for an effect of global deviancy neither on subjects' reaction times nor on accuracy. This is consistent with a previous study showing that the distraction effect of irrelevant deviant sounds decreases when task difficulty increases [53]. The three hypotheses make however different predictions regarding brain activity evoked by the visual stimulus on trials with a global auditory deviant. The first hypothesis predicts delayed and/or smaller visual activations because the deviant sound draws attention away from the visual task. The second hypothesis predicts the exact opposite, i.e. larger visual activations, because the deviant sound draws attention to the violation of the expected audio-visual chunk. The last hypothesis predicts no effect of auditory deviancy on visual activity because attentional resources would be partially independent.

**Figure 5:**
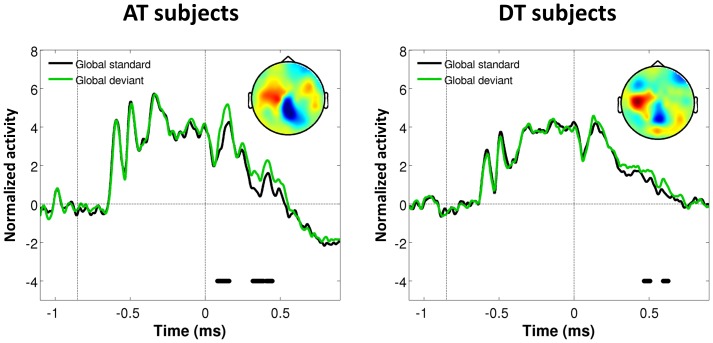
Influence of auditory violations on the visual detection task. In each panel is represented the topography (inset) and the time course of a spatial filter selective of the activity induced by the visual task. The spatial filter was computed as follow: we first subtracted Attentive ‘auditory only’ trials from Distracted ‘audio-visual’ trials, resulting in ‘purely’ visual ERFs and averaged the topographies between 500 and 650 ms after the onset of the first visual stimulus. We applied this filter to global deviant (green lines) and global standard (black lines) trials in AT (A) and DT (B) subjects in order to test whether violations of the auditory rule influenced the visual activity. Black dots show significant paired t-tests performed at each time sample, comparing ERFs amplitudes for global standard to global deviant.

We tested these three hypotheses by examining how the presentation of the deviant sound would influence visual ERFs. In order to isolate visual evoked magnetic responses, we used a recently developed spatial filtering method [47] (see method). Compared to an arbitrary selection of MEG sensors, this technique has the advantage of being data-driven and results in an optimal filter selective of visual activity. The first step was to subtract activity linked to auditory stimulation from the activity induced by ‘audio-visual’ trials (i.e. ‘audio-visual’ trials – ‘auditory only’ trials). We then selected a late component (500–650 ms) linked to the onset of the first colored ring and used the average topography as a spatial filter. Figure 5 presents the time courses of the spatial filter which revealed a stronger activation for visual stimuli when a global deviant sound was presented in both AT and DT subjects. Importantly this effect was observed in a time range comparable to the auditory global effect. For AT subjects, significant effects were observed between 82 and 160 ms, and between 318 and 448 ms. For DT subjects, the difference became significant between 467 and 507 ms, and between 594 and 628 ms (all p<0.05, figure 5). Thus, the results support the second hypothesis according to which subjects learnt a contingency between vision and audition, and detected the violation of a multimodal auditory-visual chunk.

## Discussion

In the present experiment, we manipulated subjects' attention while they were exposed to an auditory regularity. They were then presented inter-mixed trials in which sounds were either predictable when presented in parallel to a visual task or unpredictable when presented in isolation. Our predictions were that distracting subjects' attention would strongly reduce the brain's response to global violations while the response to local violations would remain essentially unaffected. As expected, the early mismatch response could be observed in both attentive and distracted trials, and was essentially driven by transitional probabilities within a sequence of sounds. On a longer time scale, we discovered that, contrary to our original predictions, the brain could still detect globally deviant sounds while performing a concurrent visual task. We suggest that the brain was able to make inferences and predictions specific to the context in which the auditory stimuli were presented [54], [55]. The processing stages indexed by late novelty responses would be flexible enough to learn different predictions for the very same auditory stimulus based on the visual context.

The GNW theory proposes that a stimulus is first integrated at the sensory level before getting access to a distributed fronto-parietal network [41]. If the subject's attention is engaged in a demanding concurrent task, the sensory information is not able to access the neuronal workspace [39]. In support to this theory, previous research using auditory paradigm similar to ours showed that the global effect actually vanished when subjects were engaged in a demanding visual task [12]. Why then did we observe a global effect on distracted trials in both groups of subjects? The main difference between the present experiment and previous ones is the timing between sequences of sounds and the distracting task: in Bekinschtein's experiment, visual and auditory stimuli occurred at random times and were completely uncorrelated. Here, such manipulation was incompatible with our goal because we wanted to mix distracted and attentive trials. Thus, we included visual and auditory stimuli in the same epochs. However, the partial temporal correlation between the two types of stimuli made it possible for attention to spill from the visual to the auditory modality [56]. Subjects might have built an audio-visual ‘chunk’, linking the colored rings to the sounds because of their close temporal proximity. In support to this hypothesis, we found visual ERFs with larger amplitudes when a deviant sound was presented.

Studies of the temporal synchrony in multisensory integration have shown that the process by which the brain evaluates the simultaneity of two sensory events tolerates a temporal jitter of a few tens of milliseconds (for review [52]). This is compatible with the temporal gaps between auditory and visual stimuli in our experiment, ranging from 0 to 250 ms (see figure 1). Thus, subjects might have learnt a contingency between vision and audition, allowing them to detect deviant ‘audio-visual’ stimuli while performing the visual task.

A second of our original predictions was that only subjects who could attend to the sounds during the exposure phase would learn the global auditory rule and show a response to global deviancy in attentive trials. Contrary to this prediction, we found differences between global standard and deviants in attentive trials neither in AT nor in DT subjects. This result may seem surprising – until one remembers that, on attentive trials, global standard and global deviant trials were equiprobable. Again, this feature of the design was indispensable to prevent DT subjects from learning the rule on attentive trials. However, it made it possible for subjects to learn a higher-order rule, with distinct auditory expectations depending on the presence or absence of a visual stimulus, even though attentive and distracted trials were intermixed. The results indeed indicate that the predictions the brain made for the upcoming stimulus were different in the two types of trials. This suggests that, in both groups of subjects, two rules related to the same stimulus were maintained in memory throughout an experimental block: their brains registered that auditory stimuli are predictable if visual stimuli are present, and unpredictable if visual stimuli are absent.

Our findings raise interesting questions regarding the impact of the history of trials on late brain components. Past studies revealed that the amplitude of the P300 component of the ERPs is directly influenced by previous trials both on a long and short temporal scale. The more a stimulus has been repeated, the larger will be the P300 component of the ERPs in response to the violation of this pattern [57], [58], [59]. If we consider that the ERFs observed here are the magnetic equivalents of the P300 (a fair assumption since a recent study using simultaneous EEG/MEG recordings and a similar local-global paradigm found that the P300 coincided with ERFs highly similar to the ones observed here (Wacongne et al., 2011)), our results show that the impact of past trials is conditioned by the context in which the stimulus was presented. The prediction the brain can make for an upcoming stimulus depends on the context and can be completely different from one context to another.

Interestingly, the examination of attentive trials in AT subjects revealed a progressive change in the amplitude of the global effect. We found larger amplitude of the brain response to the global deviant compared to the global standard at the beginning of the test phase. This difference progressively vanished until that both categories of stimuli were ultimately undistinguishable. Given the influence of stimulus probability on the P300 [58], [60], [61], it suggests that AT subjects first made predictions according to the inferences they built during the exposure phase but then adapted to the new probabilities specific to auditory-only trials, independently of the regularity in audio-visual trials. By contrast, in DT subjects, responses to global standard and global deviant stimuli were highly similar all along the test phase, showing that subjects were not biased by the stimuli presented during the exposure phase. The global effect observed on distracted trials shows that these subjects were actually able to infer a rule from the exposure phase, but they were not able to use it on attentive trials.

In sharp contrast with these highly flexible variations in global violation effects, the mismatch response induced by local violations remained unaffected by the visual task. In our paradigm, the differences between local standard and local deviant stimuli were similar in all attentional conditions and essentially driven by local transitional probabilities. As we already mentioned, previous studies showed that the MMN can be observed in inattentive conditions [11], [12], [13], [14] or in non-conscious patients [12], [13], [15], [16], [17], [18], [19], [20], [21]. However, there is also evidence that the MMN can be modulated by several factors such as attention for instance [11], [14], [62], [63] or by the history of trials [64], [65], [66].

The brain sources of the MMN have been located not only in primary and secondary auditory areas [12], [33], [63], [67] but also in the inferior frontal gyrus [68], [69], [70]. There is evidence that the frontal component of the MMN occurs later than the temporal one and might reflect a switch of attention to the deviant sound [68], [71]. Importantly, contrary to the temporal component, the amplitude of the frontal component has been observed to increase with smaller deviancy [72] and is influenced by attentional load [73]. Moreover, a lesion of the frontal cortex can decrease the amplitude of the MMN without precluding it [74]. A study also reported that the amplitude of the early part of the MMN (90–160 ms) decreased with increasing asynchrony between stimuli while the amplitude of the late part of the MMN (160–220 ms) did not [26]. Other studies showed that even if the MMN can be evoked by sequence patterns longer and more complex than the one used in our paradigm (e.g. [75]), the length of the sequence is still a limiting factor [76]. Thus, beyond the modulation of MMN under various experimental manipulations, it seems that at least part of the process can be deployed automatically and that the time range over which it can extract regularities is limited.

In our paradigm, in order to detect a violation of the global regularity, the brain would have to compare a particular sequence to past trials, i.e. over tens of seconds or even minutes. This might exceed the time window over which MMN-generating processes can extract regularities. This interpretation is compatible with recent empirical and simulated evidence which suggest that predictive coding is the main mechanism behind this mismatch response [32], [33]. In this framework, the primary auditory cortex learns the temporal statistical dependencies linking the stimuli presented in the last few seconds. These predictions are directly compared to the sensory inputs, and if the input does not match the internal model, then a prediction error signal (the MMN) is generated.

An alternative interpretation proposed by the AERS/CHAINS model [4], [77], [78] would be that MMN-related processes could actually extract both local and global regularities (at least to some extent) but one of these representations would be inhibited via competitive mechanisms. The present results are compatible with both interpretations and further research is needed to determine whether the global regularity was inhibited or never extracted at all by the MMN-related processes. Nevertheless, we show here that only brain processes linked to the extraction of the global regularity were influenced by the context in which sounds were presented.

Although the temporal range over which MMN-related processes can extract a regularity could be limited to few seconds, there is evidence that the memory trace of the regularity can last tens of seconds [79], [80]. Even when subjects are distracted, e.g. by reading a book, a regularity can be ‘reactivated’ by the presentation of a stimulus conforming to the rule [80]. A possible interpretation of this phenomenon is that if a regularity is unused, irrelevant, or if time has passed since the last presentation, the representation enters a ‘dormant’ state in which it is no longer activated but can be reinstated if a ‘reminder’ stimulus is presented [80]. According to the AERS/CHAIN model, the inhibited representation is not completely removed but rather weakened by the competition and can thus be reactivated. Alternatively, Wacongne and colleagues' model of predictive coding [32], [33] suggests that the statistical information of the sensory input is reflected in the adaptation of the synaptic weights between memory neurons and predictive subpopulations. This information coded by the synaptic weights can last several seconds and is progressively weakened with elapsed time. If a stimulus conforming to the rule is presented, then the regularity is reactivated.

In conclusion, our study shows that the process by which the brain extracts regularities from the environment is much more sophisticated than we expected. The results suggest that at a low-level (MMN), predictions are mainly driven by local transitional probabilities between stimuli while at a higher level (P300) the brain is able to make flexible inferences and predictions for a given stimulus depending on the context in which it was presented. This process is flexible enough to allow independent updating of different regularities despite inter mixed trials. Perhaps the most stimulating aspect of the present study is its potential to inspire new experimental manipulations on the relationships between a sequence of stimuli and the context in which it is presented.
